# My journey with COVID-19

**DOI:** 10.1016/j.eclinm.2020.100599

**Published:** 2020-10-17

**Authors:** Monica Malta

**Affiliations:** 1 Faculty of Medicine, Department of Psychiatry, University of Toronto, Toronto, Ontario, Canada; 2 Institute for Mental Health Policy Research, Centre for Addiction and Mental Health, Toronto, Ontario, Canada

I'm a global health researcher working to address health and gender inequalities in the Global South. During my work in areas where Malaria or Dengue Fever are endemic, I always took extra precautions to avoid getting infected. I never anticipated that while living in a large, urban city from Canada I would be at higher risk… Until the COVID-19 pandemic.

During lockdown, like most working mothers, I became the major responsible for childcare and housework [1]. To finish all my research related activities, I frequently worked until late at night. During the day I was juggling work, home and homeschooling… In mid May I started feeling weak and had more trouble breathing. As someone with an immunodeficiency disorder, I didn't pay too much attention. I though it was due to sleep deprivation and excessive working hours… But it was COVID-19. The symptoms worsened quickly and in a few days I was not able to get out of bed. Now I was under lockdown, unable to work or look after my kids, with stress piling up.

My physician considered the symptoms mild, recommending isolation and rest at home… I laughed: How does someone isolate and rest with three little kids at home and so much work to do? I was bedridden for three weeks, with difficulty breathing, headache, conjunctivitis, sore throat, aches and pain. I completely lost my appetite. During two months I could not taste or smell anything, hot or cold, sweet, salty, spice, nothing at all. My fatigue was debilitating. More than four months later, my symptoms have not gone away. My heart still races a few times a day - even while I am sitting at the computer and writing this piece. It is hard to concentrate for long periods. Imagine a scientist that cannot concentrate properly… That's me. What about the stress? It keeps piling up, with no light on the horizon.

I'm what has been identified as a ‘long-hauler’, those individuals who survived a COVID-19 infection but are enduring long-term symptoms [2]. Fatigue is one of the most common symptom, while many report racing heartbeat, enduring achy joints and shortness of breath. Several 'long-haulers’ are also experiencing long term loss of sense of smell/taste and cognitive effects often described as ‘brain fog’. To a smaller extent, some experience damage to the heart, lungs, kidneys, and brain [3]. Less than a year with this pandemic, it is still too soon to say if ‘long-haulers’ will develop a chronic disability. However, it is also way too soon to understand how long my COVID-19 damage will last. The same uncertainty is faced by hundreds of thousands of people all over the world.

The likelihood of someone with COVID-19 to develop persistent symptoms is still hard to predict, and it is unclear why it impacts some people more severely than others. But we know it is happening. A study in Italy identified that only 12.6% patients hospitalized for acute COVID-19 were completely free of any COVID-19–related symptom two months later, while 32% had 1 or 2 symptoms and 55% had 3 or more [4]. Data from the “COVID Symptom Study” includes information from over 4200,000 people using their app in the United States, United Kingdom, and Sweden. The study suggests that 10% to 15% of people don't recover within 1 month [5]. So far, it is not possible to know for how long those symptoms will endure, and whether COVID-19 might prompt the onset of chronic diseases among a subsample of patients.

Many persons experiencing long lasting COVID-19 symptoms received a negative test, and cannot prove they were ever infected in the first place [6]. For those who tested positive for COVID-19, several were not considered sick enough to be hospitalized. A very common complain of ‘long haulers’ is that some healthcare professionals dismiss their complains and symptoms, suggesting that it's just stress or anxiety [7]. The additional mental distress of dealing with health professionals disbelief while facing an unknown prognosis is brutal. Many are finding support in online groups such as the Body Politic, where more than 14,000 persons from all over the world discuss their COVID-19 symptoms - many lasting for more than 3 months [8].

Like many long-haulers, my goal is to resume previous normal and productive life. However, I still experience a plethora of long-term symptoms, including extreme fatigue and brain fog. Many long-haulers fear unemployment, as they face incapacitating symptoms and/or lower productivity. The burden of having a possible long-term condition is very stressful. But when allied with the prospects of unemployment, and for many, loss of health insurance, it can be unbearable. I'm grateful for being alive, still employed and living in a country offering universal, publicly funded healthcare. Many don't have the same privilege.

The COVID-19 pandemic is deepening inequality among the most vulnerable communities worldwide. In the Global South, a majority of HIV, TB and Malaria programs are facing disruptions, while extreme poverty and malnutrition is rising [9]. The true effect of the pandemic on mothers and infants increased mortality will only become clear in a few years [10]. If a percentage of those millions surviving COVID-19 develop long term disabilities, the burden will, once again, be heavier among highly disenfranchised populations living in the Global South.

As a researcher and a survivor, it is my duty to fight for their lives.

## Role of the funding source

NA.

## Declaration of Competing Interest

No competing interest to declare.
